# Recent infective endocarditis research findings suggest dentists prescribe prophylactic antibiotics for patients having a bicuspid aortic valve or mitral valve prolapse

**DOI:** 10.4317/medoral.25984

**Published:** 2023-06-18

**Authors:** Arthur H Friedlander, Paulo H Couto-Souza

**Affiliations:** 1 DMD. Professor-in-Residence. Oral and Maxillofacial Surgery, UCLA Dental School, Los Angeles California; 2 DMD. Director of Quality Assurance. Hospital Dental Service, Ronald Regan UCLA Medical Center, Los Angeles California; 3 DDS, PhD. Oral and Maxillofacial Surgeon and Full Professor. Department of Dentistry, School of Medicine and Life Sciences, Pontifical Catholic University of Paraná, Curitiba, Brazil

## Abstract

**Background:**

The scientific validity of the European Society of Cardiology’s (ESC) infective endocarditis (IE) guidelines limiting provision of prophylactic antibiotics (AP) only to patients having cardiac anomalies (e.g., prosthetic valves) believed to place them at “high risk” of adverse events when undergoing high risk dental procedures (HRDP) is unclear.

**Material and Methods:**

A systematic review of studies conducted between 2017 and 2022 and catalogued in the PubMed database was undertaken to ascertain if this edict was associated with changes in IE incidence, development of infection in unprotected cardiac anomalies, developing infection and resultant adverse clinical outcomes.

**Results:**

Retrieved were 19 published manuscripts, however of these, 16 were excluded because they did not bare upon the issues of concern. Among the three studies eligible for review were those in the Netherlands, Spain, and England. The results of the Dutch study denoted a significant increase in the incidence of IE cases over the projected historical trend (rate ratio: 1327, 95% CI 1.205-1.462; *p*<0.001) after the introduction of the ESC guidelines. The findings from the Spanish study evidenced the uniquely high in-hospital IE associated fatality rates suffered by patients having bicuspid aortic valves (BAV); 5.6% or mitral valve prolapse (MVP); 10%. The British study provided evidence that the incidence of fatal IE infection was significantly greater among an “intermediate risk” cohort of patients, (a group likely including those with BAC and MVP for which the ESC guidelines don’t recommend AP), than among “high risk” patients (*P* = 0.002).

**Conclusions:**

Patients having either a BAV or MVP are at significant risk of developing IE and suffering serious sequelae including death. The ESC guidelines must reclassify these specific cardiac anomalies into the “high risk” category so that AP are recognized as being needed prior to provision of HRDP.

** Key words:**Infective endocarditis, antibiotic prophylactic, dentistry.

## Introduction

In 1945, Northrop an oral surgeon concerned that patients having cardiac valvular anomalies were contracting infective endocarditis (IE) after exodontia conducted the first controlled experiment demonstrating that study group patients provided antibiotics (sulfathiazole) prior to exodontia evidenced oral streptococcal positive blood cultures far less often than medication naive controls (1). IE, a microbial infection of the endocardial surface of the heart, is an often fatal disease and can develop in association with the performance of high risk dental procedures (HRDP) involving manipulation of the gingival or periapical region of the teeth or perforation of the oral mucosa. The European Society of Cardiologists (ESC) in 2015 issued a set of guidelines declaring that dental patients having these procedures and harboring specific cardiac anomalies were at “highest risk” of developing IE and/or suffering from its adverse outcomes” and were in need of prophylactic antibiotics (AP) (2). However, it also stipulated that patients afflicted with other cardiac abnormalities such as a bicuspid aortic valve (BAV) or mitral valve prolapse (MVP) whom they termed at “intermediate risk” did not require AP. Dentists preparing their office’s protocol must evaluate this latter concept given the results of recent research findings which call it into question.

The IE pathological process is initiated when oral bacteria seed the bloodstream (transient bacteremia) and adhere to platelet/fibrin deposits formed over endocardial surfaces which have been damaged by high pressure turbulent blood flow resulting from congenitally malformed (e.g., BAV) or degenerated native heart valves (e.g., MVP) or prosthetic valves (3). As the bacteria proliferate within these masses called vegetations, they enlarge becoming a source of further bacteremia, cause valvular incompetence leading to congestive heart failure, and in part, may detach traveling downstream and lodge in a vascular bed such as in the brain causing an ischemic stroke or infarcts and infection in the lungs or kidney. Prolonged high dose intravenous antibiotics are the initial therapy however approximately 40% of patients go onto require valve repair or replacement with mortality rates approaching 30% within one year. Antibiotic prophylaxis (AP) prevents attachment of the bacteria onto damaged endocardium and may prevent these cardiac infections.

ESC Guidelines advocate administration of AP prior to performing HRDP only to patients having “high risk” cardiac anomalies defined as prosthetic cardiac valves or prosthetic material used for valve repair, a previous episode of IE, any type of cyanotic congenital heart disease (CHD), unrepaired CHD or CHD repaired with prosthetic material for the first 6 months or life-long if regurgitation remains. The recommendation not to provide AP to patients having other forms of valvular disease is predicated upon the organization’s determination that the severity level of adverse IE outcomes associated with these abnormalities are modest and more than offset by antibiotic side effects and the possible emergence of drug resistant microorganisms if additional patient groups are provided medications.

## Material and Methods

To address the research purpose, we designed and implemented a systematic review of English language studies published between 2017 and 2022, using the U.S National Library of Medicine PubMed database aggregated by numerous combinations of linked search terms: European Society of Cardiologists, infective endocarditis, dentistry, prophylactic antibiotics. Exclusion criteria were defined as studies that did not address our specific areas of concern.

## Results

**Figure 1:**
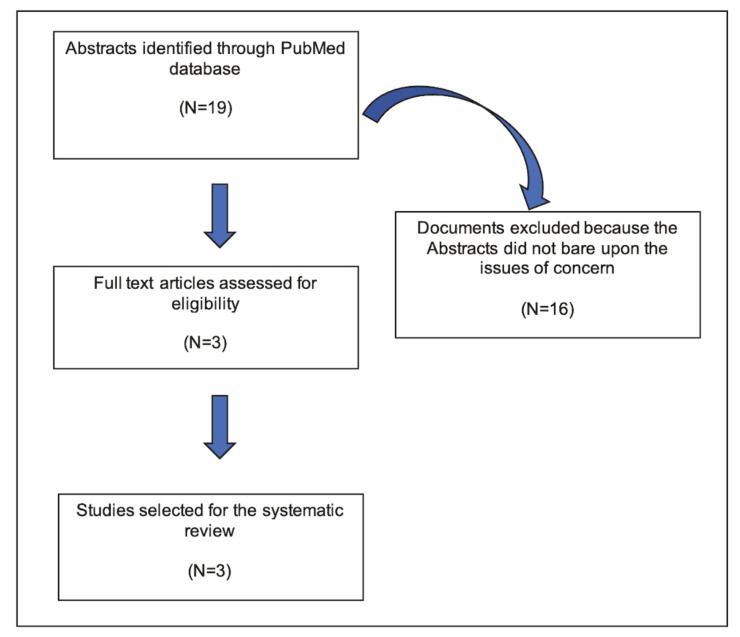
Flow chart of the systematic literature review search process.

The initial PubMed database search identified 19 abstracts for review. Invoking the exclusion criteria reduced the available documents to 3. Each of these abstracts was carefully reviewed by us (A.H.F., P.C.S.) and agreement reached that 16 reports should be excluded because they did not relate to our areas of concern (Fig. 1).

The first study emanating from Holland vividly illustrated how the ESC’s guideline changes restricting AP resulted in more patients developing the cardiac infection (4). Specifically, a nationwide retrospective trend study analyzed as an interrupted times series revealed that after operationalization of the guidelines there was a significant increased incidence of IE (rate ratio: 1327, 95% CI: 1.205-1.462; *P* < 0.001). Concomitantly, there was a related significant increase in streptococci-related IE cases (*P* = 0.0031). The results of this study are uniquely meaningful for community-based dentists because they are derived from its detailed analysis of individual patients treated by three general hospitals rather than by tertiary care centers. The authors of the study do however caution that these are observational study results and that prospective data is lacking. Thus, they concluded that to settle the debate on the relationship between prophylaxis and IE incidence prospective studies should be performed.

The second study, this one conducted by a consortium of 31 Spanish hospitals specifically devalued the ESC’s contention that patients harboring BAV or MVP are not in need of AP because the IE outcomes that they might suffer are modest (5). Among the more than 2593 consecutively admitted patients with a confirmed IE diagnosis were 1226 “high risk” patients and 1839 “low/moderate risk” patients. Within this latter group were 54 with BAV and 89 with MVP. The “low/moderate risk” cohort also termed “intermediate risk”, had a higher incidence of blood borne Viridans group streptococci from suspected odontogenic origin than that recovered from patients in the “high risk” group, and surprisingly, both groups evidenced similar rates of intracardiac complications (e.g., abscess, fistula, and perforation formation) requiring surgical management (e.g., valve repair/replacement). Even more disconcerning was that among those with BAV, 5.6% died within hospital and among those with MVP, 10% died within hospital. The results of this study demonstrate the higher than previously appreciated IE risk faced by individuals with either BAV or MVP. Thus, the authors of this study concluded that these two cardiac anomalies should be recognized in the guidelines as “high risk” conditions requiring AP.

The third study was conducted in England and gives further credence to our concerns that cohorts of patients harboring either BAV or MVP sustain IE clinical outcomes which are not benign (6). The researchers using British National Health Service data detailing all hospital admissions between 2000 and 2008 subdivided the patients having cardiac anomalies into “high” and “intermediate” IE risk cohorts and followed their progress over a minimum of 5 years to identify subsequent hospital admissions. The resultant data denoted that there were 265,436 individuals at “intermediate risk” (likely including some with BAV or MVP) of whom 3,774 (1.4%) were readmitted to hospital with an IE diagnosis and of these 943 (25%) died. Whereas among the 96,021 “high risk” individuals, 2,385 (2.5%) who were readmitted to hospital with an IE diagnosis, 508 (21%) of them died. Thus, startingly the incidence of fatal IE infection was significantly greater (*P* = 0.002) among “intermediate risk” patients than among “high risk” patients. The authors of this study emphasized that their data demonstrated that the IE risk of some “intermediate risk” patients was similar to that of several “high risk” conditions and even higher than in some with repaired congenital heart defects. They thus concluded that the guideline’s risk stratification of anomalies predisposing to IE may require re-evaluation.

## Discussion

Our interpretation of the results of these investigations is in conformance with that of each of their authors. Specifically, we and they have determined that these findings suggest that patients having either BAV or MVP should be reclassified as “high risk” and in need of AP. The implications of these findings can not be overstated and need to be widely heralded because they demonstrate that patients harboring BAV, the most common form of congenital heart disease (effecting 2% of the population) engenders a 17 fold higher incidence of IE than that observed among members of the general population. This because these valves do not open as fully as they should resulting in significant pressure gradients and turbulent blood flow which damage endocardial tissue. The resultant inflammation, fibrosis, and subsequent partial calcification enhances the susceptibility of these valves to forming infectious vegetations (7). This pathophysiologic process positions this subgroup of individuals at substatial risk of very severe IE outcomes including death (8). Similarly concerning are the IE associated fatalities sustained by patients having MVP given that this anomaly with its associated regurgitant turbulent blood flow is the most frequent predisposing cardiac condition for IE in developed countries (9-11). Furthermore, the linkage between a recent dental procedure and the development of IE in groups of patients with these two specific cardiac lesions has been substantiated by the results of other studies (12). Thus, it is quite reasonable to understand why the authors of the 3 main studies which we have identified have concluded that their research findings highlight the need for re-evaluation of the current ESC AP guidelines.

## Conclusions

Given these contemporary scientific findings we and numerous medical school-based cardiologists believe that the plausible benefits of AP for “intermediate risk” patients exhibiting BAV or MVP far exceeds the risks and therefore that there is an urgent need for the ESC to extend its 2015 guidelines such that these specific cohorts of patients is provided AP prior to HRDP (13,14). Such critical scientific information cannot be ignored by members of our profession because it raises both clinical and ethical concerns. Specifically problematic are that the ESC’s guidelines relegate to secondary importance the needs of these vulnerable patients population to society’s potential benefits of decreasing opportunities for antibiotic resistance. Furthermore, the abrogation of a specific dentist’s responsibility “to cause no harm” to the unique patient in their chair we believe is of a higher calling and consistent with the Hippocratic Oath. For it is patently obvious to us that if AP is provided to “intermediate risk” patients harboring a BAV or MVP prior to a HRDP some of these individuals will be safe guarded from the ravages of IE.
